# Magnetic β-Cyclodextrin Nanosponges for Potential Application in the Removal of the Neonicotinoid Dinotefuran from Wastewater

**DOI:** 10.3390/ijms21114079

**Published:** 2020-06-07

**Authors:** Sebastián Salazar, Nicolás Yutronic, Paul Jara

**Affiliations:** Department of Chemistry, Faculty of Science, Universidad de Chile, Las Palmeras 3425, Ñuñoa, Santiago 7800024, Chile; nyutroni@uchile.cl

**Keywords:** cyclodextrin nanosponges, inclusion compounds, magnetic nanoparticles, adsorption, nano-sorbents, water treatment

## Abstract

This article describes the use of β-cyclodextrin-based carbonate nanosponges (NSs) decorated with superparamagnetic Fe_3_O_4_ nanoparticles to study and investigate the potential removal of dinotefuran (DTF) from wastewater. The NS-DTF inclusion compound was characterized by transmission electron microscopy (TEM), energy-dispersive spectroscopy (EDS), UV-visible spectroscopy (UV-VIS), scanning electron microscopy (SEM), thermogravimetric analysis (TGA), X-ray powder diffraction (XRPD) and proton nuclear magnetic resonance (^1^H-NMR). The adsorption efficiency of NSs was evaluated as function of different contact times. The results confirmed that the NSs have a favourable sorption capacity for the chosen guest, as the polymers exhibited a maximum adsorption of 4.53 × 10^−3^ mmol/g for DTF. We also found that magnetic NSs show good reusability as they maintain their efficiency after eight adsorption and desorption cycles. Our studies and characterization by means of SEM, TEM, EDS, vibrating sample magnetometer (VSM) and UV-VIS also show that NSs with magnetic properties are excellent tools for insecticide removal from aqueous environments.

## 1. Introduction

Neonicotinoids are a class of insecticides with widespread use in crop production and pest control. The neonicotinoid family includes insecticides such as imidacloprid, acetamiprid, dinotefuran (DTF), and thiamethoxam, among others. Neonicotinoids present a low risk for non-target organisms and the environment, as they are highly specific for subtypes of nicotinic receptors that occur primarily in insects [1,2]. However, as the DTF mechanism of action involves disruption of the insect nervous system by inhibiting nicotinic acetylcholine receptors, neonicotinoids might be a potential threat to beneficial insects such as honeybees [3]. Honeybees are essential for ecosystems, as they contribute to pollination for most crop species and wild flowering plants [4]. Numerous studies have indicated a correlation between honeybee welfare and neonicotinoid exposure, which has disastrous effects on honeybee performance and survival. Much of the crisis in honeybee populations has been attributed to the use of neonicotinoids [5,6]. In view of this concern, advances in nanotechnology adsorption could be of great value [7,8,9,10].

Among pollutant removal techniques, cyclodextrin (CD)-based carbonate nanosponges (NSs) stand out, as such polymers have a well-defined structure, moderate toxicity when administered orally and the ability to form inclusion complexes [7,8,9]. Nanosponges have also been reported to be excellent substrates for the deposition of nanoparticles, such as Fe_3_O_4_ nanoparticles, thus improving the efficiency and properties of the NSs [10].

Magnetite nanoparticles have also attracted considerable attention because of their potential applications. Superparamagnetic nanoparticles are known to be great when it comes to water purification, as they have a great surface-to-volume ratio and are easy to handle by means of an external magnetic field [10,11]. These nanomaterials also present good stability and reusability, especially after their deposition on the surface of an organic polymer [10,11].

The objectives of this study were to use β-cyclodextrin NSs decorated with Fe_3_O_4_ nanoparticles to investigate their sorption properties for DTF, as these polymers have been reported to have great potential for the sequestration of organic compounds such as pesticides [10,11,12,13,14,15,16,17,18,19], drugs [20,21,22,23,24] and dyes [25,26]. Native β-CDs have previously been used to improve the sorption efficiency of neonicotinoid on nanomaterials such as graphene and metal organic frameworks [27,28,29]. This study contributes to the understanding of the potential applications of NSs in terms of neonicotinoid removal from the environment.

## 2. Results and Discussion

### 2.1. Characterization of NSs Decorated with Fe_3_O_4_ Nanoparticles

**Figure 1:**
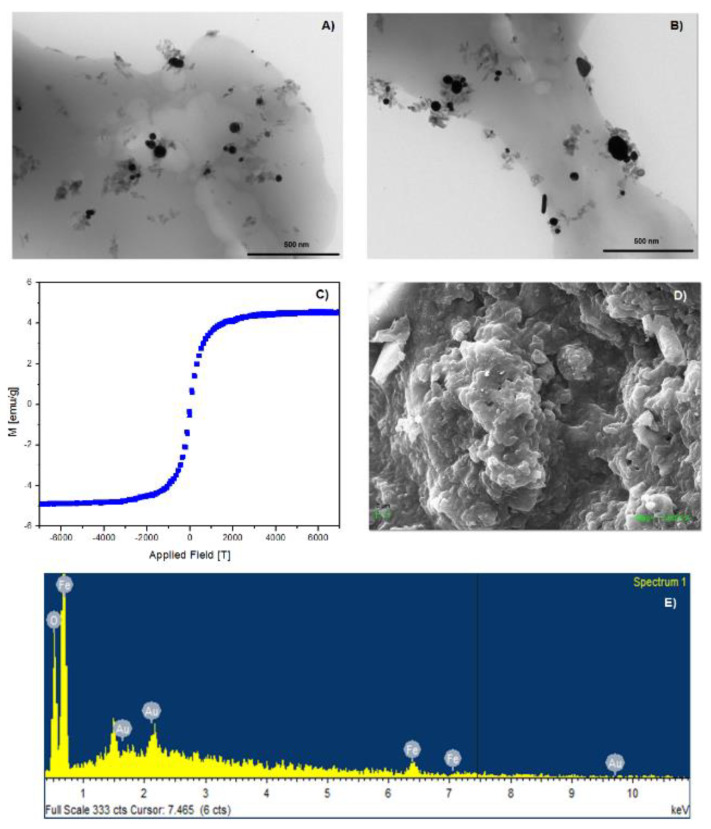
TEM; (**A**,**B**) VSM; (**C**) SEM; (**D**) and EDS; (**E**) analyses of NSs decorated with Fe_3_O_4_ nanoparticles.

Magnetic NS characterization was carried out by means of VSM, TEM, SEM, and EDS. The analyses are shown in Figure 1. Fe_3_O_4_ nanoparticles were successfully deposited on the surface of NSs, and the nanoparticles retained their superparamagnetic properties after being immobilized on the polymer. As shown in Figure 1A,B, magnetite nanoparticles are deposited uniformly on the surface of the NSs, thus preventing their agglomeration. VSM analysis at room temperature shows that the value of magnetization saturation is 5 emu/g. The graph also shows that remanence and coercivity values are close to zero, confirming the superparamagnetic nature of the magnetite nanoparticles after deposition on the surface of NSs. The SEM analysis in Figure 1D shows that NSs retain their morphology and porous structure after decoration with magnetite nanoparticles. EDS, in Figure 1E, provided elemental composition and weight percentage of iron and oxygen, confirming the deposition of magnetite nanoparticles on NSs. Au presence is due to sputter coating.

### 2.2. H-NMR Spectra of NS-DTF Inclusion Compound

**Figure 2:**
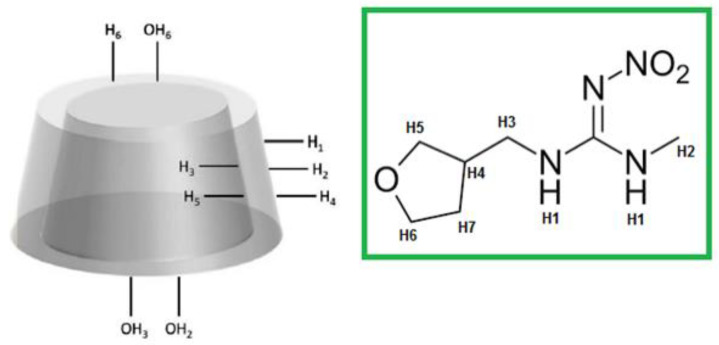
Proton assignments for the CD monomer and DTF.

The formation of the NS-DTF inclusion complex was confirmed using ^1^H-NMR spectroscopy. Proton assignments for DTF and the CD monomer are shown in Figure 2. The proton signals from DTF showed high-field chemical shifts due to the spatial restriction of the guest as it is included inside the cavities of NSs. The H3 and H5 signals also showed chemical shifts, which confirmed inclusion, as these signals correspond to the internal protons of the NSs. These results are consistent with previous studies with native CDs and NSs [10,30,31,32,33,34].

The chemical shifts for the protons of DTF after its inclusion inside NSs are shown in Table 1.

**Figure 3:**
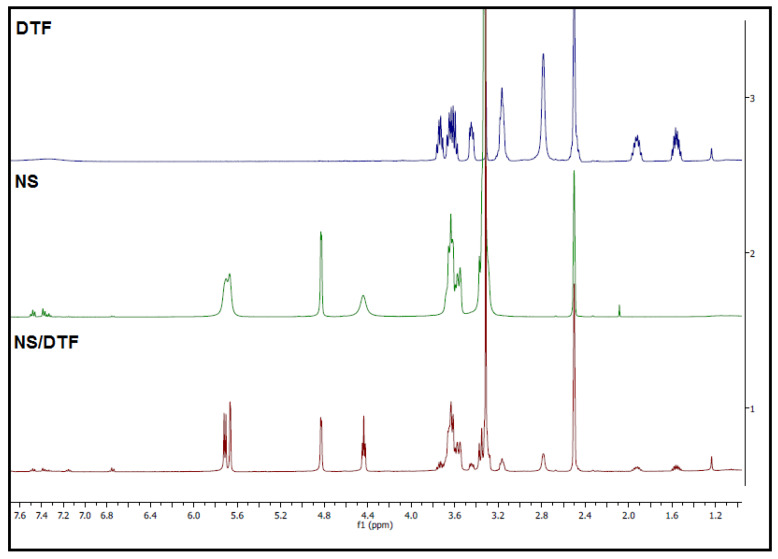
^1^H-NMR spectra (400 MHz, DMSO-d^6^) of DTF, NS and the NS–DTF complex.

^1^H-NMR spectra for the NSs–DTF complex are shown in Figure 3. As NSs are insoluble in deuterated water/chloroform, deuterated DMSO was used as a solvent for the ^1^H-NMR analyses. Previous studies have shown that DMSO does not interfere with the formation of the inclusion compound [10,30,31,32,33,34]. As seen in Figure 3, ^1^H-NMR spectra show that some of the signals in the NS–DTF complex are sharper than that of NSs alone. This could be due to the inclusion of DTF not only inside the cavities of the CDs monomers, but also inside the interstices of NSs. These results are consistent with our previous studies using cyclodextrin-based carbonate NSs with different guests [10] and with recent studies [33].

### 2.3. XRPD of the NS–DTF Inclusion Compound

**Figure 4:**
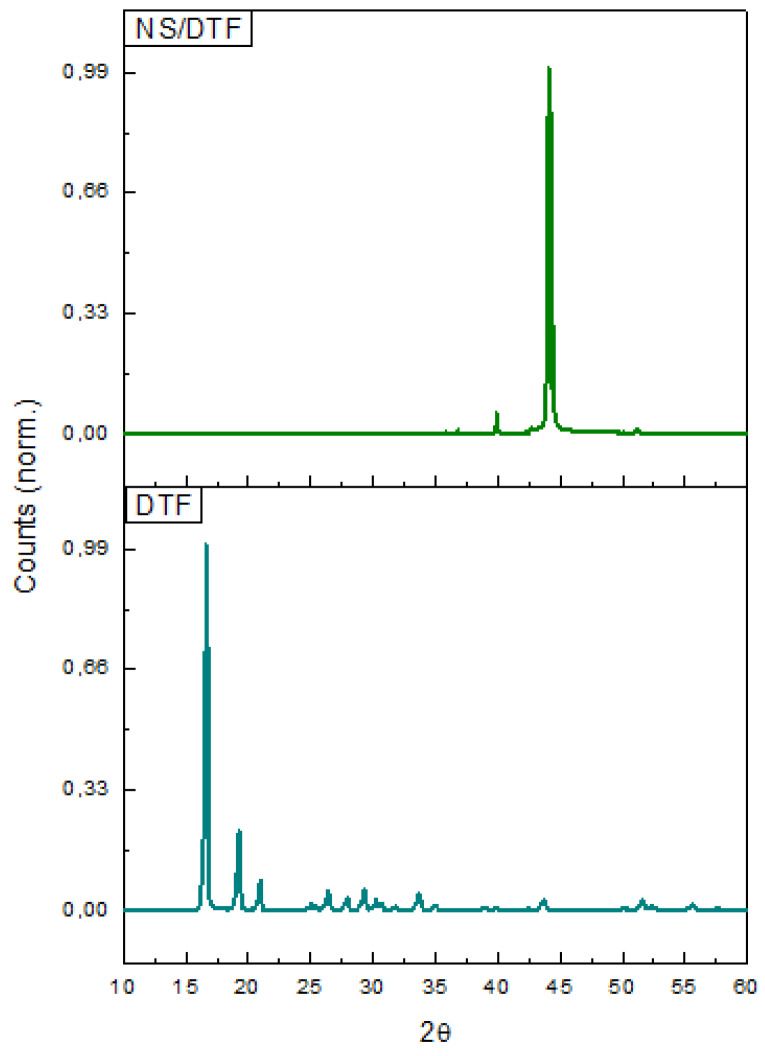
XRPD analysis of DTF and the NS–DTF inclusion complex.

The XRPD analysis of DTF and NS–DTF is shown in Figure 4. The XRPD diffractograms confirm the formation of the inclusion compound, as most of the DTF characteristic peaks are reduced in the NS/DTF diffraction pattern. The diffractograms also confirmed the lack of free DTF and native CD in the inclusion compound.

### 2.4. TGA of the NS-DFT Inclusion Compound

**Figure 5:**
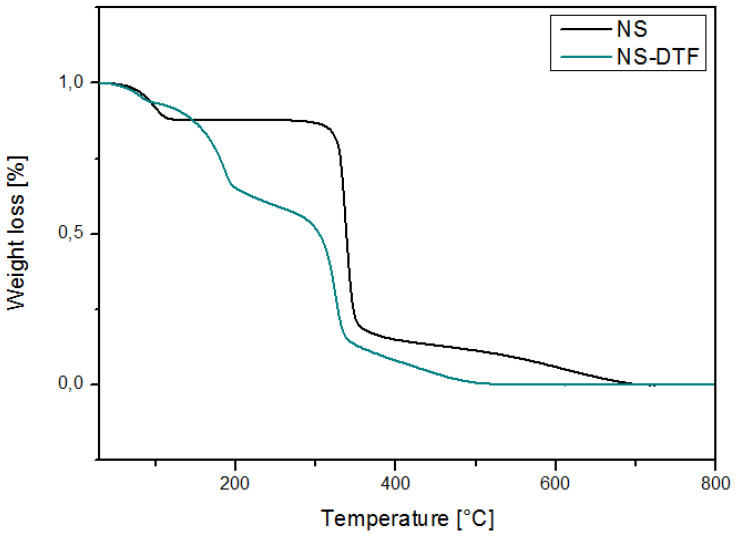
TGA analysis of NS and the NS–DTF complex.

Figure 5 shows the TGA analysis of NSs and the NS–DTF complex. Weight loss occurred at 100 °C due to the loss of water molecules in both NSs and the NS–DTF complex. The complexes started to degrade at 83 °C, followed by the main degradation step at 300 °C. The NS–DTF thermogram shows weight loss at 185 °C, which corresponds to the degradation of DTF. The inclusion of DTF in the cavities of the NSs might slightly increase the thermal stability of the guest.

Decomposition temperatures of NSs and the NS–DTF complex are summarized in Table 2. Decomposition occurs in two distinct steps for NSs and in three mass-loss steps for the NS–DTF complex.

### 2.5. SEM and EDS of the NS–DTF Complex

**Figure 6:**
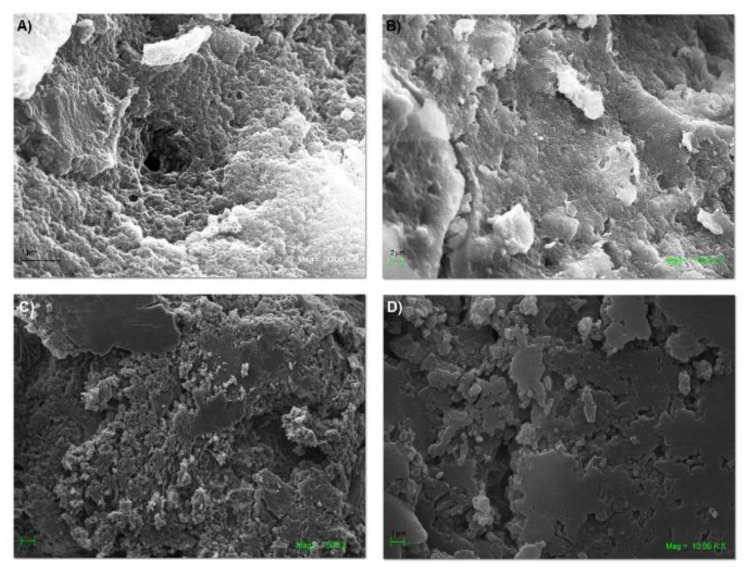
SEM analysis of NSs: (**A**,**B**) and the NS–DTF inclusion compound (**C**,**D**).

The formation of the inclusion compound was also confirmed by SEM and EDS analyses. Figure 6 shows changes in the morphology of the NSs, as most of their pores were filled with the pesticide guest.

**Figure 7:**
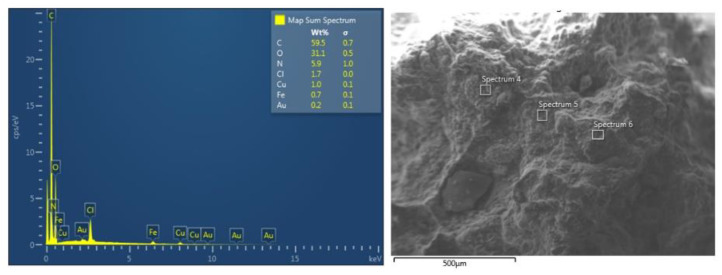
EDS spectrum of the NS–DTF complex.

The EDS analysis is shown in Figure 7. This analysis provides information about the elemental composition of the inclusion compound, which confirmed the presence of DTF in the cavities of the NSs. The graph shows the weight percentages and presence of carbon, oxygen and nitrogen, the latter element corresponding to the amine, imine and nitro functional groups of DTF. The iron presence is due to the magnetite nanoparticles deposited on the surface of NSs. Au presence is due to sputter coating.

### 2.6. TEM of the NS–DTF Complex

**Figure 8:**
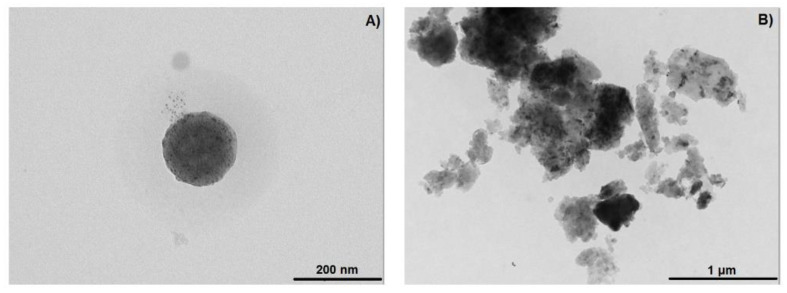
TEM analysis of: NSs (**A**) and the NS–DTF inclusion compound (**B**).

Figure 8 shows TEM analyses of plain NSs and the NS–DTF complex. TEM micrographs show that plain NS are spherical in shape and reveal an average size of 100 nm. The NS–DTF micrographs show a higher degree of aggregation and a change in size and shape in comparison with NSs.

### 2.7. Sorption Efficiency and Loading Capacity

#### 2.7.1. Molar Attenuation

The molar attenuation of DTF was determined using the Lambert–Beer equation. The absorbance of eight pesticide solutions was recorded using UV-VIS spectroscopy at 290 nm. The obtained molar attenuation (**ε**) for DTF was 11.0 mM/cm.

#### 2.7.2. Sorption Efficiency

Ce was determined using the calibration curves obtained from Section 2.7.1., and Qe was determined using Equation (1). Ce and Qe values are shown on Table 3.

**Figure 9:**
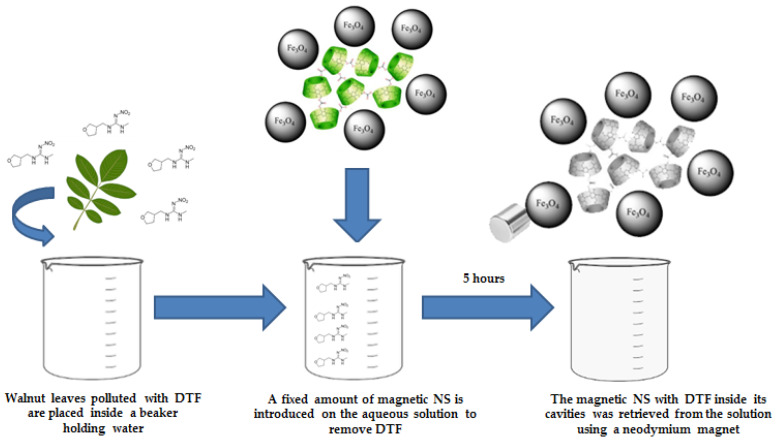
Schematic representation of DTF removal and retrieval of magnetic NSs.

Figure 9 shows a schematic representation of DTF removal with magnetic NSs with the retrieving of NSs by the use of an external magnetic field as well. Walnut (*Juglans Regia*) leaves impregnated with DTF were placed in a beaker. Magnetic NSs were introduced in the same beaker and retrieved afterwards using a neodymium magnet (5000 gauss, 25 × 20 mm) in order to demonstrate their ability to remove the insecticide as well as their reusability. The values of VSM were 5 emu/g, which is enough to retrieve the magnetic NSs from the media [35,36]. For the experiment, the solution volume was 10 mL, the concentration of DTF was 0.01 mM, and the amount of NSs used was 20 mg, previously decorated with 10 mL of magnetic nanoparticles.

**Figure 10:**
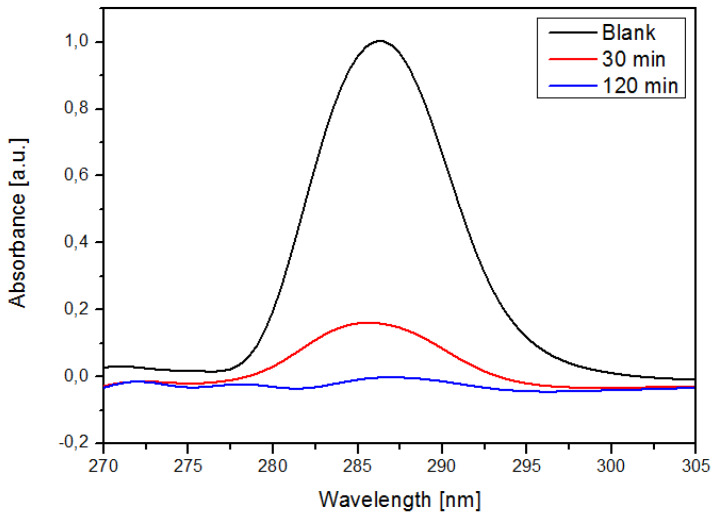
UV-VIS spectra of DTF after different contact times with NS.

UV-VIS analysis was carried out in order to determine the sorption efficiency of NS. Figure 10 shows that the max absorbance of DTF decreased as the contact time with NS increased, thus confirming that NS are excellent tools when it comes to the removal of neonicotinoids.

The maximum absorbance of DTF decreased as the contact time with NSs increased. UV spectral analysis showed that NSs are viable materials for the removal of DTF.

**Figure 11:**
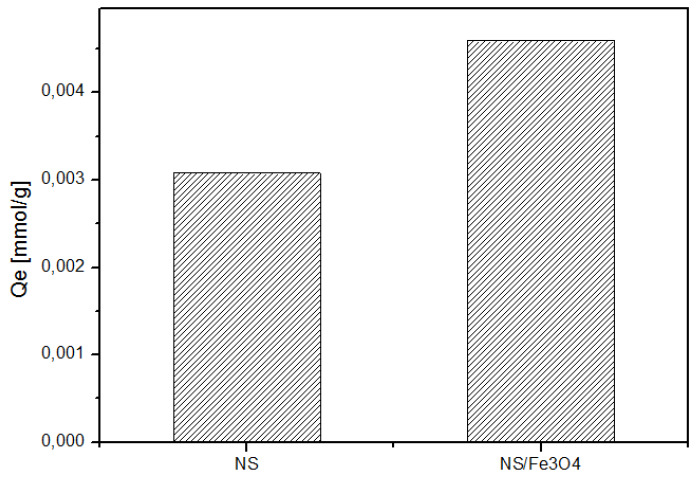
Amount of DTF adsorbed with plain NSs and magnetic NSs.

Figure 11 shows the loading capacities of native NSs and magnetic NSs. Loading capacities were as follows: NS/Fe_3_O_4_ > NSs. The results also show that magnetic NSs show improved sorption capacity after being decorated with magnetic nanoparticles and their sorption efficiency is similar to those of nanomaterials such as functionalized graphene composites (r-GO) [37].

### 2.8. Reutilization of Magnetic NSs

**Figure 12:**
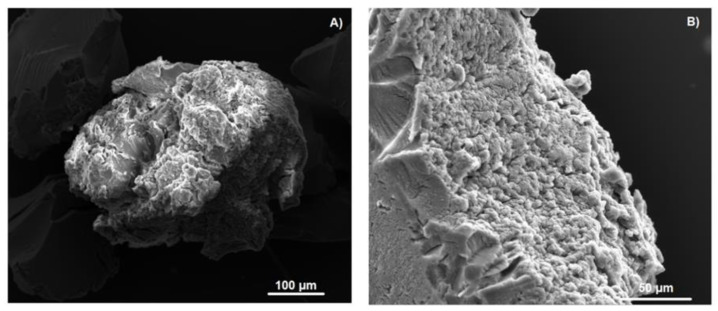
SEM images of magnetic NSs after a repeated number of cycles (**A**,**B**).

Reutilization of magnetic NSs was studied by performing repetitive adsorption experiments using the same polymer. Magnetic NSs were regenerated by extraction in a Soxhlet apparatus with acetone and milli-Q water. SEM micrographs in Figure 12 show that the surface of magnetic NSs changed drastically after the repeated cycles as the pores were filled with the DTF guest.

**Figure 13:**
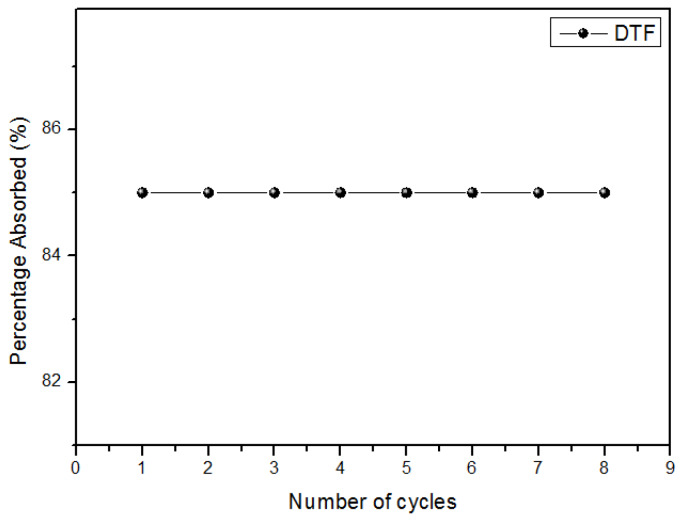
Percentage of DTF absorbed after a repeated number of cycles.

Figure 13 shows that magnetic NSs maintain their effectiveness after repeated cycles, thus proving that the polymer is a cost-effective material.

## 3. Materials and Methods

### 3.1. Materials

All chemical reactants used in this study are commercially available and were used as received: β-CD (Sigma-Aldrich, Saint Louis, MO, USA), DTF (Merck, Darmstadt, Germany), diphenylcarbonate (DPC) (Sigma-Aldrich, Saint Louis, MO, USA) and Milli-Q water (Merck, Darmstadt, Germany). Glassware used for the experiments was washed with aqua regia (HCl and HNO_3_ at a molar ratio of 3:1) and then rinsed repeatedly with Milli-Q water.

### 3.2. Synthesis of Fe_3_O_4_ Nanoparticles

Fe_3_O_4_ nanoparticles were synthetized by the co-precipitation method, as reported previously [10,38,39]. Solutions of 0.1 M FeCl_2_ × 4H_2_O (1 mL) and 0.2 M FeCl_3_ × 6H_2_O (4 mL) were prepared in 0.1 M HCl in a round-bottom flask under argon gas supply to maintain an inert atmosphere. Precipitation was performed with dropwise addition of 50 mL of 1 M NH_3_ under stirring (pH 9.7). The reaction mixture was stirred until a black-coloured colloidal solution was formed. The ferrofluid was separated using a neodymium alloy magnet of 5000 G and washed using distilled water. The Fe_3_O_4_ nanoparticles were stored at 4 °C to prevent oxidation to maghemite [40].

### 3.3. Synthesis of NSs

NS synthesis was performed using a published procedure [41] with slight modifications [10]. The NSs were prepared using 1.5 g of β-CD and 0.856 g of DPC (molar ratio 1:4). Homogenized anhydrous β-CD and DPC were placed in an Erlenmeyer flask. The mixture was heated to 90 °C in an ultrasound bath and left to react for 6 h. The reaction mixture was left to cool, and the obtained white powder was broken down roughly with an agate mortar. The solid was repeatedly washed with distilled water to remove unreacted β-CD and with ethanol to remove unreacted DPC and phenol, which was a by-product of the reaction.

Afterwards, the solid was extracted in a Soxhlet apparatus with acetone for 48 h. Finally, the solid was dried at 60 °C in an oven for 48 h and stored at 25 °C for further use.

### 3.4. NS/DTF Inclusion Compound

A fixed amount (20 mg) of NSs was suspended in 10 mL of a DTF solution in a glass container and kept for 24 h under stirring at room temperature. The suspension was centrifuged at 15,000× *g* rpm for 20 min and then dried under vacuum for further use.

### 3.5. Decoration of NSs with Fe_3_O_4_ Nanoparticles

A determined mass of NSs (20 mg) was suspended in 10 mL of magnetite nanoparticles. The suspension was allowed to settle and then centrifuged at 20,000× *g* rpm for 20 min. The NSs changed from white to grey once the magnetite nanoparticles had been deposited on the polymer, with a magnetite content of 3% wt. NSs functionalized with magnetite were dried under vacuum and then exposed to a neodymium alloy magnet to evaluate the magnetic response of the polymer [10].

### 3.6. Characterization of NSs

Proton nuclear magnetic resonance (^1^H-NMR), XRPD, TGA, scanning electron microscopy (SEM), Fourier-transform infrared spectroscopy (FT-IR), Brunauer–Emmett–Teller (BET), DLS, and TEM analyses were performed to confirm the formation of NSs. Further details can be found in reference [10].

### 3.7. Characterization of Fe_3_O_4_ Nanoparticles

XRPD, TGA, transmission electron microscopy (TEM), zeta potential (Z-potential), dynamic light scattering (DLS), energy-dispersive spectroscopy (EDS), saturation magnetization, UV-visible spectroscopy (UV-VIS) and selected area electron diffraction (SAED) analyses were carried out to confirm the presence of Fe_3_O_4_ nanoparticles.

Details for the zero field-cooled (ZFC), field-cooled (FC), DLS, magnetic response, XRPD, EDS, SAED, Z-potential and TEM analyses of the Fe_3_O_4_ nanoparticles can be found in reference [10].

### 3.8. Characterization of NS/DTF Inclusion Compound

^1^H-NMR spectra of NS/DTF were obtained using a Bruker Avance 400 MHz spectrometer with 16 scans. Stock solutions of NS/DTF were prepared in deuterated DMSO [42]. The surface morphology of NS/DTF was determined using a LEO VP1400 analytical scanning electron microscope equipped with an Oxford 7424 energy-dispersive spectrometer. The NS/DTF inclusion compound was centrifuged at 12,000× *g* rpm and then dried under vacuum. Solid samples were sputter-coated with gold and prepared by the application of carbon films on aluminium stubs. UV-VIS spectra of NS/DTF were recorded using a Perkin Elmer Lambda 25 UV-VIS spectrometer. Measurements were carried out over a range of 250–500 nm using deionized water as a reference [10].

### 3.9. Characterization of NSs Decorated with Fe_3_O_4_ Nanoparticles

The saturation magnetization of NSs decorated with Fe_3_O_4_ nanoparticles was measured using a vibrating sample magnetometer (VSM) at room temperature. The surface morphology of NSs decorated with magnetite nanoparticles was determined using a LEO VP1400 analytical scanning electron microscope equipped with an Oxford 7424 energy-dispersive spectrometer [10].

### 3.10. Sorption Efficiency

Sorption efficiency was carried out using reported procedures [10,17,20]. A DTF solution (0.01 mM) was prepared at pH 7.0. A set amount of magnetic NSs (20 mg) was added to a fixed volume (10 mL) of insecticide solution in a glass container.

The container was sealed and placed in a magnetic stirrer at room temperature. DTF concentration was determined at different contact times with NSs (30–120 min) using a spectrophotometer to monitor absorbance changes.

The equilibrium concentration of DTF in the NSs (*Qe*) removed from the solution is defined by Equation (1), where *Co* is the initial insecticide concentration, *Ce* is the final insecticide concentration, *V* is the solution volume, and *m* is the polymer mass:

(1) $$Qe=\frac{\left(Co-Ce\right)V}{m}$$

## 4. Conclusions

In this work, magnetic NSs were used and characterized for the removal of DTF from the environment. NSs efficiently formed a complex with DTF. H-NMR, XRPD, TGA, SEM, EDS and UV-VIS analyses showed that NSs have favourable sorption for neonicotinoids in solution. The NSs polymers exhibited a maximum adsorption of 4.53 × 10^−3^ mmol/g for DTF. SEM, TEM, EDS and VSM analyses confirmed that NSs are excellent substrates to stabilize magnetite nanoparticles, thus giving the polymer additional properties, as magnetic NSs were easily recovered from the solution by the use of a neodymium magnet. The reusability of magnetic NSs was tested, showing 100% efficiency until the eighth sorption/desorption cycle. Magnetic NSs may eventually become an improved technology for neonicotinoid removal from aquatic environments, as they are efficient, low-cost, non-toxic and reusable materials.

## Figures and Tables

**Table 1:** Chemical shifts for DTF and NS after the formation of the inclusion compound.

H DTF	δ DTF (ppm)	δ NS–DTF (ppm)	Δδ (ppm)
H1	2.035	2.026	−0.009
H2	2.811	2.809	−0.003
H3	1.440	1.437	−0.003
H4	1.927	1.922	−0.005
H5	3.827	3.825	−0.002
H6	3.572	3.569	−0.003
H7	1.985	1.982	−0.003
**H NS**	**δ NS (ppm)**	**δ NS–DTF (ppm)**	**Δδ (ppm)**
OH 2	5.704	5.717	0.013
OH 3	5.670	5.671	0.001
OH 6	4.440	4.447	0.007
H 1	4.827	4.830	0.003
H 3	3.627	3.635	0.008
H 5	3.572	3.575	0.003
H 6	3.572	3.575	0.003

**Table 2:** Decomposition temperatures of NS and the NS-DTF complex.

Sample	First Decomposition	Second Decomposition	Third Decomposition
NS	113.4 °C	344.8 °C	-
NS-DTF	115.1 °C	185.4 °C	341.7 °C

**Table 3:** Ce and Qe values for DTF at different contact times with NSs.

Sample	Contact Time [min]	Ce [mM]	Qe [mmol/g]	Uptake
DTF	30	2.83 × 10^−3^	3.59 × 10^−3^	71.7%
DTF	120	9.65 × 10^−4^	4.53 × 10^−3^	90.3%

## Abbreviations

DMSO	Dimethyl Sulfoxide
NSs	Nanosponges
β-CD	Beta Cyclodextrin
DTF	Dinotefuran
^1^H-NMR	Proton nuclear magnetic resonance
TGA	Thermogravimetric analysis
XRPD	X-ray powder diffraction
SEM	Scanning electron microscope
TEM	Transmission electron microscope
VSM	Vibrating sample magnetometer
EDS	Energy Dispersive Spectroscopy
DPC	Diphenylcarbonate
